# Superiority of Minimally Invasive Oesophagectomy in Reducing In-Hospital Mortality of Patients with Resectable Oesophageal Cancer: A Meta-Analysis

**DOI:** 10.1371/journal.pone.0132889

**Published:** 2015-07-21

**Authors:** Can Zhou, Li Zhang, Hua Wang, Xiaoxia Ma, Bohui Shi, Wuke Chen, Jianjun He, Ke Wang, Peijun Liu, Yu Ren

**Affiliations:** 1 Department of Breast Surgery, the First Affiliated Hospital of Xi’an Jiaotong University, Xi’an, Shan’xi Province, China; 2 Department of Translational Medicine Center, the First Affiliated Hospital of Xi’an Jiaotong University, Xi’an, Shaanxi Province, China; Baylor College of Medicine, UNITED STATES

## Abstract

**Background:**

Compared with open oesophagectomy (OE), minimally invasive oesophagectomy (MIO) proves to have benefits in reducing the risk of pulmonary complications for patients with resectable oesophageal cancer. However, it is unknown whether MIO has superiority in reducing the occurrence of in-hospital mortality (IHM).

**Objective:**

The objective of this meta-analysis was to explore the effect of MIO vs. OE on the occurrence of in-hospital mortality (IHM).

**Data Sources:**

Sources such as Medline (through December 31, 2014), Embase (through December 31, 2014), Wiley Online Library (through December 31, 2014), and the Cochrane Library (through December 31, 2014) were searched.

**Study Selection:**

Data of randomized and non-randomized clinical trials related to MIO versus OE were included.

**Interventions:**

Eligible studies were those that reported patients who underwent MIO procedure. The control group included patients undergoing conventional OE.

**Study Appraisal and Synthesis Methods:**

Fixed or random -effects models were used to calculate summary odds ratios (ORs) or relative risks (RRs) for quantification of associations. Heterogeneity among studies was evaluated by using Cochran’s Q and I^2^ statistics.

**Results:**

A total of 48 studies involving 14,311 cases of resectable oesophageal cancer were included in the meta-analysis. Compared to patients undergoing OE, patients undergoing MIO had statistically reduced occurrence of IHM (OR=0.69, 95%CI =0.55 -0.86). Patients undergoing MIO also had significantly reduced incidence of pulmonary complications (PCs) (RR=0.73, 95%CI = 0.63-0.86), pulmonary embolism (PE) (OR=0.71, 95%CI= 0.51-0.99) and arrhythmia (OR=0.79, 95%CI = 0.68-0.92). Non-significant reductions were observed among the included studies in the occurrence of anastomotic leak (AL) (OR=0.93, 95%CI =0.78-1.11), or Gastric Tip Necrosis (GTN) (OR=0.89, 95%CI =0.54-1.49).

**Limitation:**

Most of the included studies were non-randomized case-control studies, with a diversity of study designs, demographics of participants and surgical intervention.

**Conclusions:**

Minimally invasive oesophagectomy (MIO) has superiority over open oesophagectomy (OE) in terms of the occurrence of in-hospital mortality (IHM) and should be the first-choice surgical procedure in esophageal surgery.

## Introduction

Surgical resections remain the mainstay of potentially curative treatment for resectable oesophageal cancer [1–6]. However, resections for esophageal cancer are invasive, and various surgical techniques for open oesophagectomy (OE) have been considered to have high mortality and morbidity rates [7]. Previous studies found that the occurrence of in-hospital mortality was between 1.2 and 8.8% [7–11], even as high as 29% [12]. Therefore, in-hospital mortality has often been considered as an outcome indicator for esophageal surgery and used to analyze and compare surgical outcomes among different medical centres [13]. Therefore, the exploration for measures to prevent in-hospital death and relevant factor are the hotter and more discussed issues in current studies of esophageal surgery, and any achievement in this aspect may have a deep impact on the clinical treatment of oesophageal cancer.

Minimally invasive oesophagectomy (MIO), first described in 1990s [14–16], has superiority in reducing the risk of postoperative morbidity without compromising oncological outcomes through avoiding thoracotomy and laparotomy [4, 17–20]. Theoretically, MIO has an advantage over OE in reduction the risk of IHM to a larger extent. Nevertheless, this theoretical assumption has never been subjected to empirical verification [20–33]. Instead, previous meta-analyses [22–33], relevant studies [4–6] and even randomized controlled trials [3] of available evidences have suggested a potential advantage of MIO in reducing the incidence of morbidity, rather than in reducing mortality.

Thus, at least two critical questions concerning esophageal surgery are of considerable interest and remain unanswered: i) does MIO have superiority in reducing the occurrence of IHM?; ii) what are the factors affecting the occurrence of IHM? These questions are important for both future research and current clinical practice. For this reason, we conducted a systematic review and meta-analysis to comprehensively assess the superiority of MIO in reducing the occurrence of IHM, with the aim to provide meaningful clues for esophageal surgery.

## Methods

### Data sources and searches

Medline (through December 31, 2014), Embase (through December 31, 2014), Wiley Online Library (through December 31, 2014), and the Cochrane Library, (through December 31, 2014) were searched, using the terms “Minimally invasive oesophagectomy, oesophageal cancer, oesophageal carcinoma,open oesophagectomy”. This review protocol was registered and published in the International Prospective Register of Systematic Reviews, PROSPERO (Registration No. CRD42014012901), following the prescribed steps [34]. This report complies with the preferred reporting items for systematic reviews and meta-analyses (PRISMA) [35–36].

### Study Selection and Eligibility Criteria

Included studies had to meet the following criteria: i) research articles published in English; ii) randomized or non-randomized controlled studies with parallel controls; iii) studies comparing MIO with OE; iv) grey literature such as conference proceedings, reports and other peer-reviewed research.

Publications were excluded: i) if the outcomes of interest was not reported or it was impossible to calculate the outcomes from the published results; ii) if a distinct group of patients was not mentioned or the outcomes of interest were not compared; iii) if publications belong to systematic reviews or meta-analysis.

### Data collection and Quality Assessment

All eligible studies were retrieved and evaluated by two independent reviewers. Disagreements on inclusion were discussed, if necessary, with the guidance of the corresponding authors of these studies via E-mail. If no response was received, a second E-mail was sent one week later.

To ascertain the validity of eligible studies, study quality was appraised in reference to the 12 items described in methodological index for non-randomized studies (MINORS) [37]. The total quality scores ranged from 0 (low quality) to 24 (high quality). Disagreement on study quality was resolved by discussion with corresponding authors of these studies via E-mail or personal interview.

### Outcomes Definition

IHM was defined as hospital mortality, inpatient mortality, mortality within 30 days of hospitalization, in-patient death, death in hospital, or mortality. The broad definition of MIO was thoracoscopic/laparotomy assisted oesophagectomy, hybrid minimally invasive oesophagectomy and total thoracoscopic/ laparoscopic oesophagectomy, or minimally invasive oesophagectomy (MIE). Pulmonary complications were defined as respiratory complications, pulmonary infection, pneumonia, respiratory failure, adult respiratory distress syndrome, atelectasis, etc., but did not include adult respiratory distress syndrome. Arrhythmia was defined as atrial arrhythmia or atrial fibrillation.

### Data synthesis and analysis

In-hospital mortality was the primary outcome measure, as it was considered an outcome indicator for esophageal surgery and has been used to analyze and compare surgical outcomes among different medical centres. Secondary outcome measures included pulmonary complications, pulmonary embolism, anastomotic leak, gastric tip necrosis, and arrhythmia, for the reason that they are underlying causes of in-hospital mortality. Fixed or random-effects models [38] were used in this meta-analysis. Forest plots were provided to illustrate pooled relative risks (RRs) or odds ratios (ORs), and corresponding 95% confidence intervals (CIs). The consistency of results (effect sizes) among studies was investigated by means of I^2^ statistics [39]. When the heterogeneity test was statistically significant, a random effects model was used, otherwise, a fixed effects model was used. Heterogeneity was interpreted according to the thresholds outlined in the Cochrane Handbook: 0% to 40%- low heterogeneity, 30% to 60%- moderate heterogeneity, 50% to 90%-possible substantial heterogeneity, 75% to 100%- considerable heterogeneity. If the heterogeneity was high [40] (I^2^>50% or P<0.10), sensitivity analysis and subgroup analysis were performed to find out potential origin of heterogeneity.

Egger's test and Begg’s funnel plot were used for diagnosis of potential publication bias [41]. In addition, the possible effect of publication bias in our meta-analysis was further assessed using Duval and Tweedie nonparametric “trim and fill” procedure [42]. This method considers the possibility of hypothetical “missing” studies that might exist, imputes their RRs, and recalculates a pooled RR that incorporates the hypothetical “missing” studies as though they actually existed.

All statistical processes were performed with Stata version 12.0 software (Stata Corp LP, College Station, TX, USA).

## Results

### Selected studies and methodological quality

**Fig 1:**
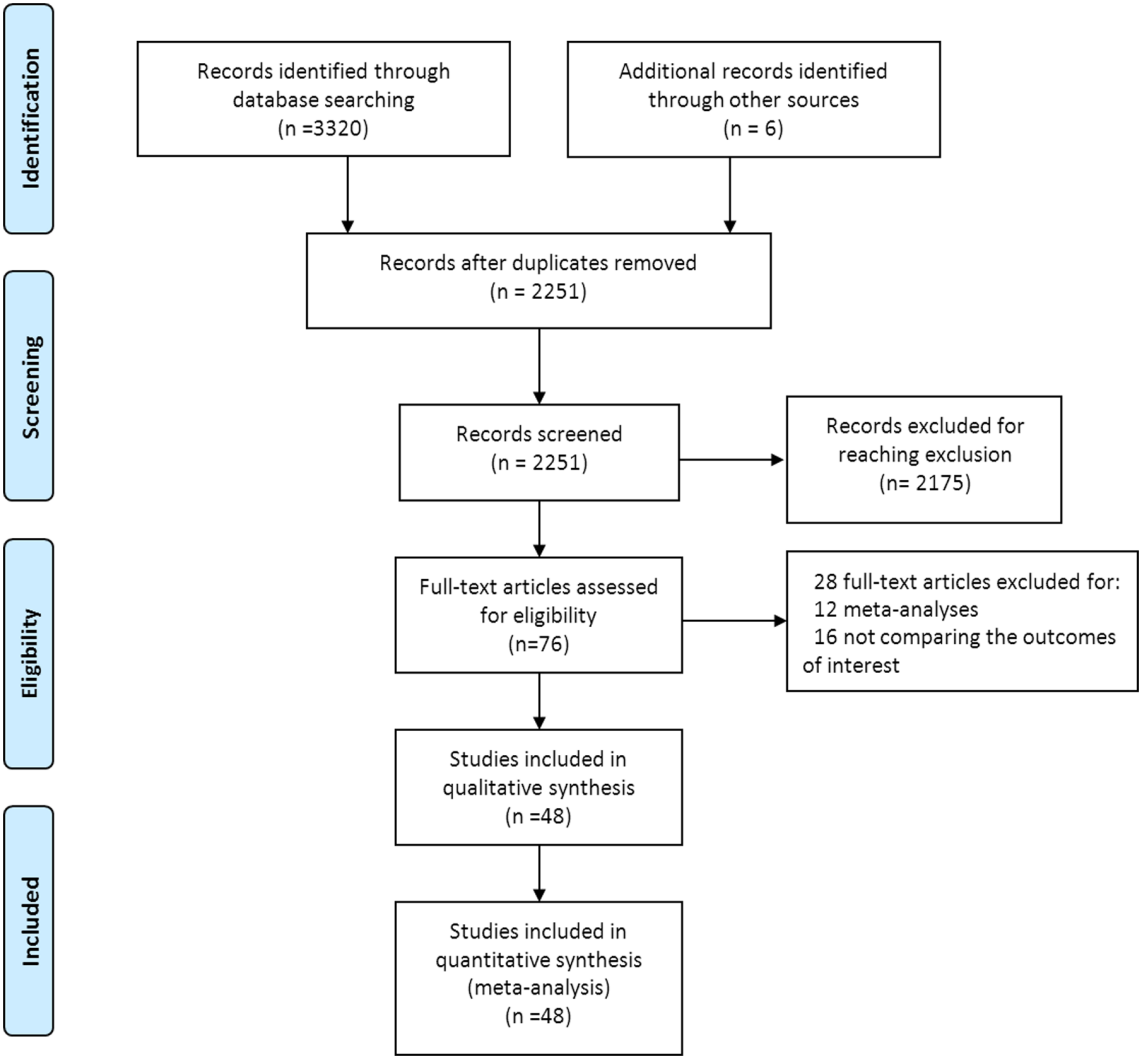
Flow Diagram of the search and selection method.

The steps of our literature search are shown in Fig 1. A total 3,326 unique records were identified from the electronic databases. Of these, 1,075 records were excluded for duplicated ones, 2,175 records were excluded for meeting the exclusion criteria. After an initial screening of titles and abstracts, 76 potential articles were included for full-text view [3, 4, 6, 21–33, 43–102]. Twenty-eight articles were excluded after additional screening, with the reasons that: i) 12 studies were meta-analyses or systematic overviews [22–33]; ii) 14 studies did not compare the outcomes of interest [89–102]; iii) 2 studies were retrieved from the same registry [53,62] and contained an overlapping group of patients with the recent publications [75,78]. Thus, in total, 48 articles with 14,311 patients undergoing MIO versus OE were included in this systematic review and meta-analysis.

The evaluation results of the methodological quality of the studies are shown in Table 1. The quality scores of the included studies ranged from 16 to 20 (Table 1). None of the included studies performed a prospective calculation of the study size or an unbiased assessment of study outcomes. A randomized controlled design was done in only one study [4].

**Table 1:** Characteristics and Demographics of Included Studies.

Study	Year	Country	Design	Cases		MINORS	Adeno.%	Hybrid	TNM stage			
				MIO	OE				MIO		OE	
									0+I+II	III+IV	0+I+II	III+IV
Law S^[43]^	1997	China	NA	18	63	16	NA	MIE	5	13	15	48
Nguyen NT^[44]^	2000	USA	R	18	36	16	NA	MIE	NA	NA	NA	NA
Osugi H^[45]^	2003	Japan	P	77	72	20	0	TA	NA	NA	NA	NA
Kunisaki C^[46]^	2004	Japan	P	15	30	16	NA	MIE	NA	NA	NA	NA
Van den Broek WT^[47]^	2004	Netherlands	NA	25	20	19	71.1	TA	8	17	10	10
Braghetto I^[48]^	2006	Chile	R	47	119	20	NA	MIE	41	6	29	90
Bresadola V^[49]^	2006	Italy	NA	14	14	16	NA	MIE	11	3	6	8
Shiraishi T^[50]^	2006	Japan	R	116	37	16	NA	Hybrid	NA	NA	NA	NA
Benzoni E^[51]^	2007	Italy	P	9	13	20	23.8	TA	9	0	7	6
Smithers BM^[3]^	2007	Australia	P	332	114	20	70.6	Hybrid	192	118	36	75
Fabian T^[52]^	2008	USA	R	22	43	20	69.2	MIE	14	7	25	19
Parameswaran R^[54]^	2009	UK	NA	50	30	19	82.5	MIE	27	23	17	15
Perry KA^[55]^	2009	USA	P	21	21	16	45.2	MIE	NA	NA	NA	NA
Saha AK^[56]^	2009	UK	NA	16	28	19	16+	MIE	NA	NA	NA	NA
Zingg U^[57]^	2009	Australia	NA	56	98	20	72.1	MIE	35	21	47	42
Hamouda AH^[58]^	2010	UK	P	51	24	16	80	MIE	NA	NA	NA	NA
Pham TH^[21]^	2010	USA	P	44	46	16	74.4	MIE	20	20	20	19
Safranek PM^[59]^	2010	UK	P	75	46	16	NA	Hybrid	31	44	29	17
Schoppmann SF^[60]^	2010	Australia	P	31	31	20	46.8	MIE	14	15	13	17
Schröder W^[61]^	2010	Germany	R	238	181	16	60.1	TA	NA	NA	NA	NA
Wang H^[63]^	2010	China	NA	27	29	16	5.3	TA	24	3	24	5
Berger AC^[64]^	2011	USA	NA	65	53	16	79.7	MIE	52	13	41	12
Gao Y^[65]^	2011	China	R	96	78	16	5.2	MIE	54	42	40	38
Lee JM^[66]^	2011	Japan	P	74	64	20	5.1	Hybrid	45	20	49	15
Nafteux P^[67]^	2011	Belgium	R	65	101	16	75.3	MIE	NA	NA	NA	NA
Yamasaki M^[68]^	2011	Japan	R	109	107	20	NA	TA	NA	NA	NA	NA
Briez N^[69]^	2012	France	P	140	140	20	40.7	TA	92	48	89	51
Kinjo Y^[70]^	2012	Japan	P	106	79	19	3.2	MIE	65	41	45	34
Maas KW^[71]^	2012	Netherlands	R	50	50	20	69	MIE	19	31	19	31
Mamidanna R^[72]^	2012	UK	P	1155	6347	16	NA	MIE	NA	NA	NA	NA
Sihag S^[73]^	2012	USA	P	38	76	16	85.1	MIE	29	9	53	23
Sundaram A^[74]^	2012	USA	R	47	57	20	78.8	MIE	NA	NA	NA	NA
Tsujimoto H^[75]^	2012	Japan	NA	22	27	16	NA	TA	9	11	19	18
Bailey L^[76]^	2013	UK	P	39	31	20	82.9	TA	NA	NA	NA	NA
Biere SS^[4]^	2013	Netherlands	RCT	59	56	18	61.7	MIE	31	15	26	19
Ichikawa H^[77]^	2013	Japan	NA	153	162	20	66.7	TA	101	51	81	79
Kitagawa H^[78]^	2013	Japan	R	45	47	16	4.3	MIE	NA	NA	NA	NA
Noble F^[79]^	2013	UK	P	53	53	19	NA	MIE	NA	NA	NA	NA
Parameswaran R^[80]^	2013	UK	P	67	19	16	75.6	Hybrid	43	23	8	11
Takeno S^[81]^	2013	Japan	R	91	166	20	3.5	TA	NA	NA	NA	NA
Kubo N^[6]^	2014	Japan	R	135	74	16	NA	Hybrid	112	23	41	33
Mu J^[82]^	2014	China	R	176	142	16	NA	MIE	34	18	73	17
Kauppi J^[83]^	2014	Finland	R	74	79	20	NA	MIE	28	46	25	54
Meng F^[84]^	2014	China	R	94	89	20	4.4	MIE	56	38	50	39
Schneider C^[85]^	2014	UK	R	19	61	16	78.8	MIE	16	2	36	24
Zhang J^[86]^	2014	China	R	60	61	16	NA	MIE	NA	NA	NA	NA
Li J^[87]^	2014	China	R	89	318	20	NA	MIE	64	25	188	126
Javidfar J^[88]^	2012	USA	R	92	165	20	86.8	MIE	65	27	96	69

Note: Adeno.: adenocarcinoma; NA: not applicable; MIO: minimally invasive oesophagectomy, including MIE,TA and Hybrid MIE; OE: open esophagectomy; MIE: total minimally invasive esophagectomy; TA: thoracoscopic-assisted MIE; Hybrid: Hybrid minimally invasive oesophagectomy.

### Characteristics of studies and patients

The 48 studies totaling in 14,311 patients included in this meta-analysis contained 4,509 (30.5%) cases undergoing MIO and 9,793 (69.5%) undergoing OE (Table 1). Of the 48 studies, only 1 was a randomized controlled trial (RCT) [4]. Eight studies [54,56,58,59,72,76,79,80,85] were done in the United Kingdom (UN), 8 in the USA [21,44,52,55,64,73,74,88],11 in Japan [6,45,46,50,66,68,70,75,77,78,81], 7 in China [43,63,65,82,84,86, 87], 4 in Australia [3,57,60,67],3 in Netherlands [4,47,71], and 2 in Italy [49,51], and the remaining studies were conducted in Germany [61], France [69], Chile [48], and Finland [83]. Key methodological characteristics are shown in Table 1. Thirty-one studies investigated in-hospital mortality (IHM) as an outcome measure, 42 studies for pulmonary complications (PCs), 17 studies for pulmonary embolism (PE), 25 studies for Arrhythmia, 41 studies for Anastomotic Leak (AL) and 17 studies for Gastric Tip Necrosis (GTN) (Table 2).

**Table 2:** Outcomes of interest in Included Studies.

Study	Endpoints											
	Hospital Mortality		Pulmonary complications		Pulmonary embolism		Arrhythmia		Anastomotic Leak		Gastric Tip Necrosis	
	MIO	OE	MIO	OE	MIO	OE	MIO	OE	MIO	OE	MIO	OE
Law S^[43]^	NA	NA	3/18	11/63	NA	NA	NA	NA	NA	NA	NA	NA
Nguyen NT^[44]^	NA	NA	2/18	6/36	1/18	1/36	NA	NA	2/18	4/40	0/18	1/36
Osugi H^[45]^	NA	NA	NA	NA	12/149	3/72	2/77	2/77	1/149	2/53	NA	NA
Kunisaki C^[46]^	NA	NA	0/15	1/30	NA	NA	NA	NA	2/15	1/30	NA	NA
Van den Broek WT^[47]^	NA	NA	2/25	2/20	NA	NA	NA	NA	2/25	3/20	NA	NA
Braghetto I^[48]^	3/47	13/119	7/47	22/119	0/47	1/119	NA	NA	4/47	26/119	NA	NA
Bresadola V^[49]^	NA	NA	1/14	2/14	1/14	0/14	NA	NA	1/14	2/14	NA	NA
Shiraishi T^[50]^	6/116	5/37	25/116	12/37	NA	NA	3/116	4/37	13/116	9/56	NA	NA
Benzoni E^[51]^	NA	NA	0/8	1/13	NA	NA	NA	NA	1/8	1/13	0/8	1/13
Smithers BM^[3]^	7/332	3/114	87/332	35/114	NA	NA	55/332	21/114	18/332	10/114	5/332	2/114
Fabian T^[52]^	1/22	4/43	3/22	18/43	1/22	0/43	4/22	8/43	3/22	3/43	1/22	0/43
Parameswaran R^[54]^	NA	NA	4/50	2/30	0/50	1/30	0/50	2/30	4/50	1/30	5/50	2/30
Perry KA^[55]^	NA	NA	2/21	3/21	NA	NA	5/21	8/21	4/21	6/21	NA	NA
Saha AK^[56]^	0/16	2/28	NA	NA	NA	NA	NA	NA	2/16	3/28	NA	NA
Zingg U^[57]^	2/56	6/98	17/56	33/98	NA	NA	NA	NA	11/56	11/98	NA	NA
Hamouda AH^[58]^	NA	NA	15/51	5/24	NA	NA	3/51	6/24	4/51	2/24	3/51	1/24
Pham TH^[21]^	3/44	2/46	13/44	9/46	0/44	2/46	18/44	11/46	4/44	5/46	1/44	1/46
Safranek PM^[59]^	3/75	1/46	19/75	13/46	NA	NA	NA	NA	11/75	1/46	2/75	0/46
Schoppmann SF^[60]^	NA	NA	5/31	17/31	NA	NA	NA	NA	1/31	8/31	0/31	1/31
Schröder W^[61]^	7/238	11/181	NA	NA	NA	NA	NA	NA	18/238	17/181	NA	NA
Wang H^[63]^	NA	NA	1/27	5/29	0/27	1/29	2/27	1/29	5/27	4/29	NA	NA
Berger AC^[64]^	5/65	4/53	5/65	12/53	NA	NA	NA	NA	9/65	6/53	NA	NA
Gao Y^[65]^	2/96	3/78	13/96	11/78	NA	NA	NA	NA	7/96	6/47	NA	NA
Lee JM^[66]^	4/74	8/64	11/74	20/64	NA	NA	NA	NA	10/74	18/60	NA	NA
Nafteux P^[67]^	2/65	2/101	47/65	17/101	NA	NA	NA	NA	5/65	10/101	NA	NA
Yamasaki M^[68]^	0/109	2/107	7/109	15/107	NA	NA	3/109	6/107	6/109	4/166	0/109	2/107
Briez N^[69]^	2/140	10/140	22/140	60/140	NA	NA	NA	NA	8/140	6/140	0/140	1/140
Kinjo Y^[70]^	NA	NA	22/106	31/79	NA	NA	10/106	4/79	8/106	10/29	NA	NA
Maas KW^[71]^	0/50	1/50	9/50	13/50	NA	NA	3/50	6/50	4/50	3/50	NA	NA
Mamidanna R^[72]^	46/1155	274/6347	230/1155	1181/6347	19/1155	92/6347	102/1155	611/6347	NA	NA	NA	NA
Sihag S^[73]^	0/38	2/76	1/38	33/76	0/38	2/76	5/38	12/76	0/38	2/76	NA	NA
Sundaram A^[74]^	2/47	1/57	5/47	19/57	5/47	19/57	9/47	19/57	4/47	4/57	NA	NA
Tsujimoto H^[75]^	1/22	5/27	2/22	10/27	NA	NA	3/22	14/27	7/22	3/31	1/22	0/27
Bailey L^[76]^	2/39	2/31	15/39	18/31	NA	NA	3/39	8/31	NA	NA	NA	NA
Biere SS^[4]^	2/59	1/56	7/59	19/56	1/59	9/56	NA	NA	7/59	4/56	NA	NA
Ichikawa H^[77]^	0/153	8/153	20/153	33/153	NA	NA	17/153	38/153	14/153	27/153	4/153	5/153
Kitagawa H^[78]^	1/45	2/47	6/45	14/47	NA	NA	NA	NA	NA	NA	NA	NA
Noble F^[79]^	NA	NA	18/53	14/53	0/53	1/53	6/53	6/53	5/53	2/53	NA	NA
Parameswaran R^[80]^	3/67	1/19	NA	NA	11/67	2/19	1/67	2/19	NA	NA	9/67	2/19
Takeno S^[81]^	4/91	15/166	NA	NA	NA	NA	NA	NA	NA	NA	NA	NA
Kubo N^[6]^	2/135	2/74	13/135	16/74	NA	NA	NA	NA	10/135	7/74	0/135	2/74
Mu J^[82]^	1/176	1/142	6/176	4/142	NA	NA	NA	NA	12/176	4/142	NA	NA
Kauppi J^[83]^	NA	NA	13/74	15/79	5/74	5/79	14/74	20/79	5/74	5/79	0/74	2/79
Meng F^[84]^	1/94	4/89	9/49	24/89	NA	NA	4/94	11/89	6/94	7/89	NA	NA
Schneider C^[85]^	0/19	2/61	NA	NA	NA	NA	NA	NA	NA	NA	NA	NA
Zhang J^[86]^	NA	NA	4/60	7/61	NA	NA	3/60	5/61	3/60	2/61	NA	NA
Li J^[87]^	3/89	16/318	15/89	76/318	1/89	3/318	7/89	29/318	19/89	45/318	NA	NA
Javidfar J^[88]^	NA	NA	9/92	29/165	3/92	4/165	22/92	46/165	5/92	7/165	2/92	2/165

Note: NA: not applicable; MIO: minimally invasive oesophagectomy, including MIE, thoracoscopic-assisted MIE and Hybrid MIE; OE: open esophagectomy.

Large variations existed in the pathological types of the tumors: 32 studies reported the cases of adenocarcinoma, with the proportions ranging from 0% to 86.8%, whereas 16 studies failed to mention the pathological types. In addition, 31studies involved total MIE, 11 studies thoracoscopic- assisted MIE (TA), and 6 studies Hybrid (some cases underwent TA while some underwent MIE). In addition, TNM stage were reported in 31 studies totaling in 4440 cases, of whom 63.5% (1,346/2,119) were early stage (stage I and II) of esophageal cancer in the MIO group, and only 54.2% (1,257/2,321) were early stage in the OE group.

## Results

### MIO and Risk of In-hospital Mortality (IHM)

**Fig 2:**
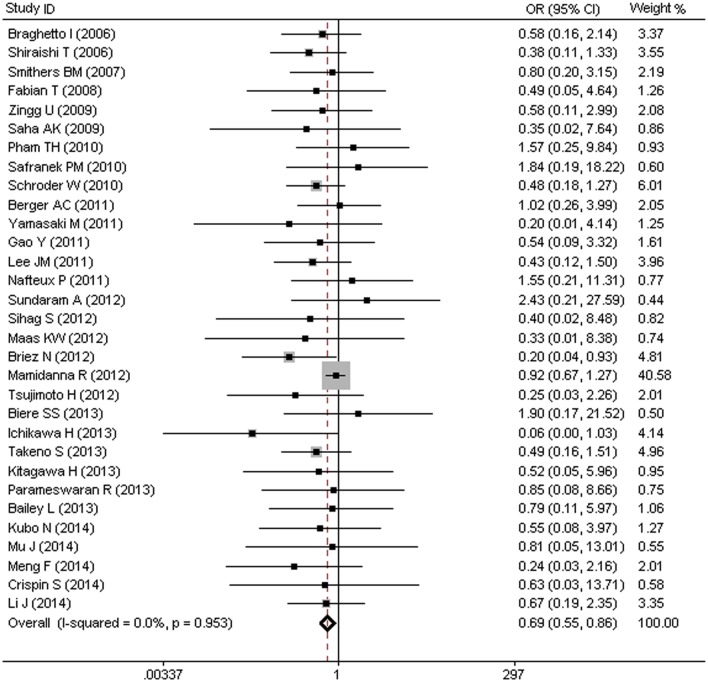
MIO and Risk of In-Hospital Mortality (IHM).

Thirty-one trials including a total of 13,117 patients were included, with an overall in-hospital mortality (IHM) rate of 4.0% (528/13,117). Of the 13,267 patients, 4.6% (413/8,968) were allocated to OE group and 3.0% (115/3,774) were allocated to MIO group, As shown in Fig 2, the pooled OR of 0.69 (95%CI = 0.55–0.86) indicated a significant reduction in the risk of IHM after treated with MIO, with no heterogeneity among the included studies (I^2^ = 0%, p = 0.953).

### MIO and Risk of Pulmonary complications (PCs)

Data for pulmonary complications (PCs) was available for 42 studies or 13,267 cases. Of the 13,267 patients included in these studies, 17.8% (715/4,006) of the patients were allocated to MIO group and 20.4% (1,888/9,261) of the patients allocated to OE group developed PCs, with an overall morbidity of 19.6% (2,603/13,267).

**Fig 3:**
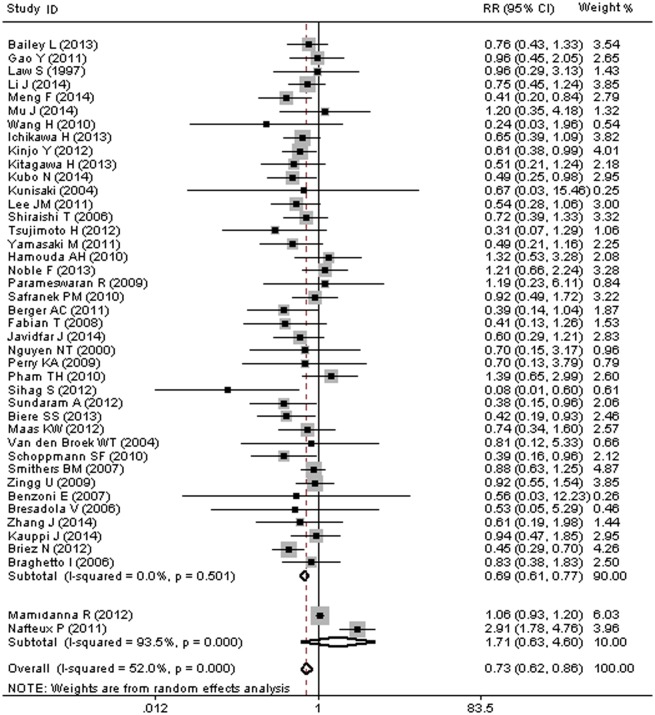
MIO and Risk of Pulmonary Complications (PCs).

As shown in Fig 3, due to a statistically significant heterogeneity (I^2^ = 52.0%, p<0.001), random-effects model as well as subgroup analysis was performed. The pooled RR of 0.73 (95%CI = 0.62–0.86) revealed a significant effect of MIO in reducing the risk of PCs. A consistent result from the subgroup analysis (RR = 0.69, 95% CI: 0.61–0.77) after removing two studies [67, 72], which might be the source of heterogeneity, demonstrated that MIO intervention was associated with a difference in the occurrence of PCs (Fig 3), with no significant heterogeneity (I^2^ = 0%, p = 0.501).

### MIO and Risk of Pulmonary Embolism (PE)

**Fig 4:**
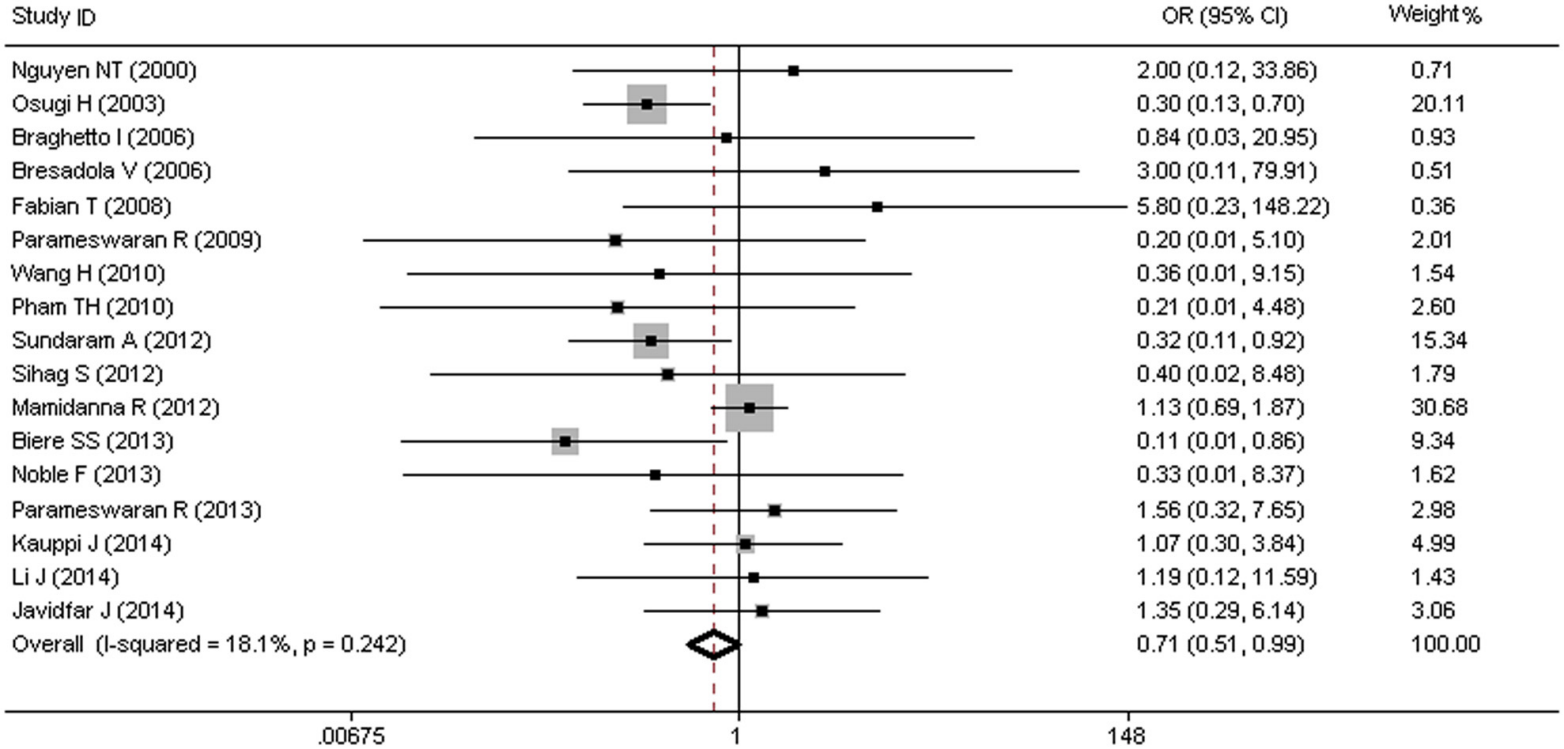
MIO and Risk of Pulmonary Embolism (PE).

Seventeen studies, including a total of 9,585 patients, evaluated the efficacy of MIO in reducing the risk of pulmonary embolism (PE). Of the 9,585 patients, 2,045 underwent OE and 7,540 underwent MIO, with an overall PE morbidity of 2.3% (217/9,585). As shown in Fig 4, the pooled OR of 0.71 (95% CI = 0.51–0.99) showed an obvious downward trend of the PE morbidity, with no heterogeneity (I^2^ = 18.1%, p = 0.242).

### MIO and Risk of Arrhythmia

**Fig 5:**
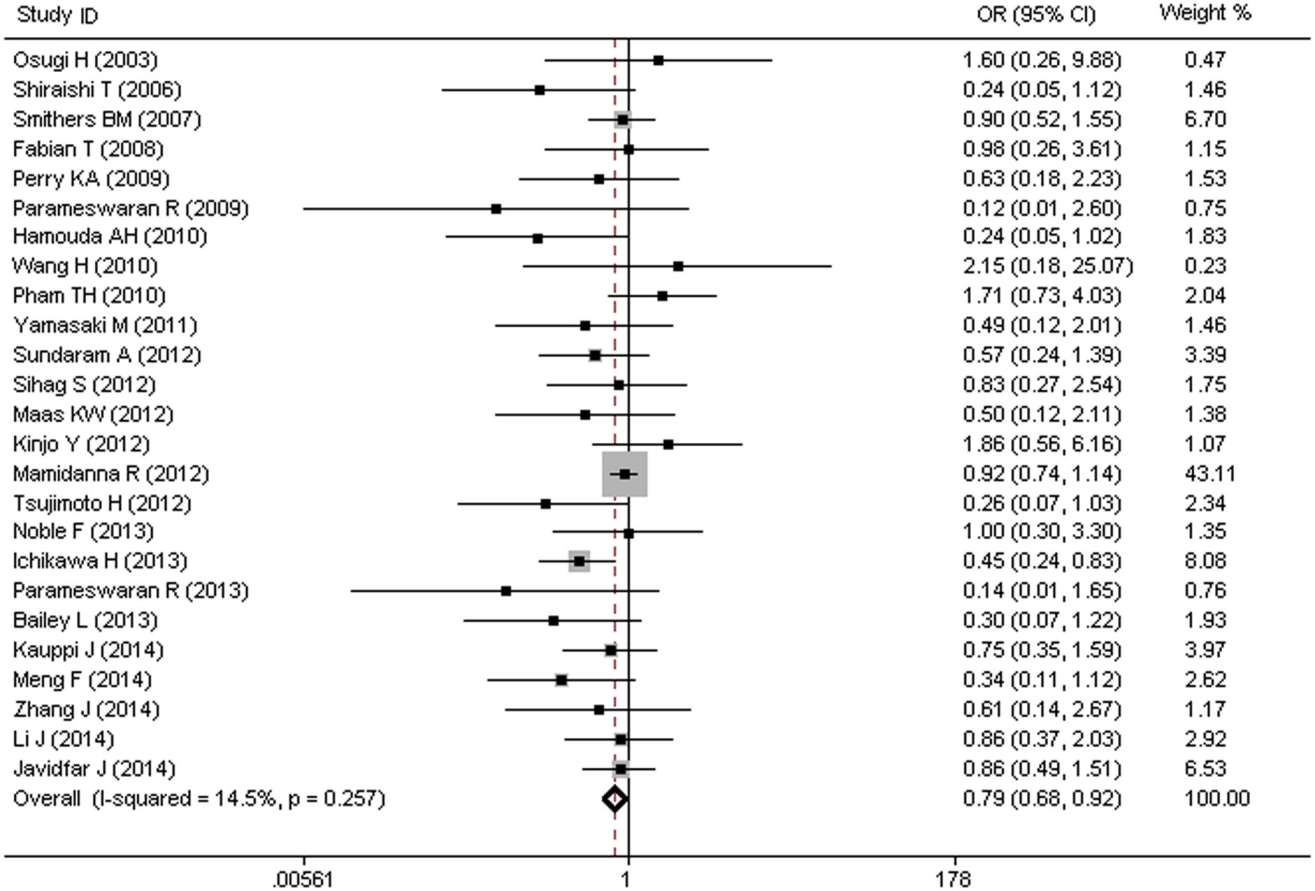
MIO and Risk of Arrhythmia.

Twenty five trials, including a total of 11,115 participants, of whom 2,983 underwent OE and 8,132 underwent MIO, evaluated the efficacy of MIO in reducing the risk of arrhythmia. Of these participants, 10.2% (305/2,983) of the patients in MIO group and 11.0% (900/8,132) in OE group developed arrhythmia, with an overall morbidity of 10.8% (1,205/11,115). It can be seen that the MIO group, as shown in Fig 5, showed a significant decrease in the morbidity of arrhythmia (OR = 0.79, 95%CI = 0.68–0.92), with no heterogeneity among different studies (I^2^ = 14.5%, P = 0.257).

### MIO and Risk of Anastomotic Leak (AL)

**Fig 6:**
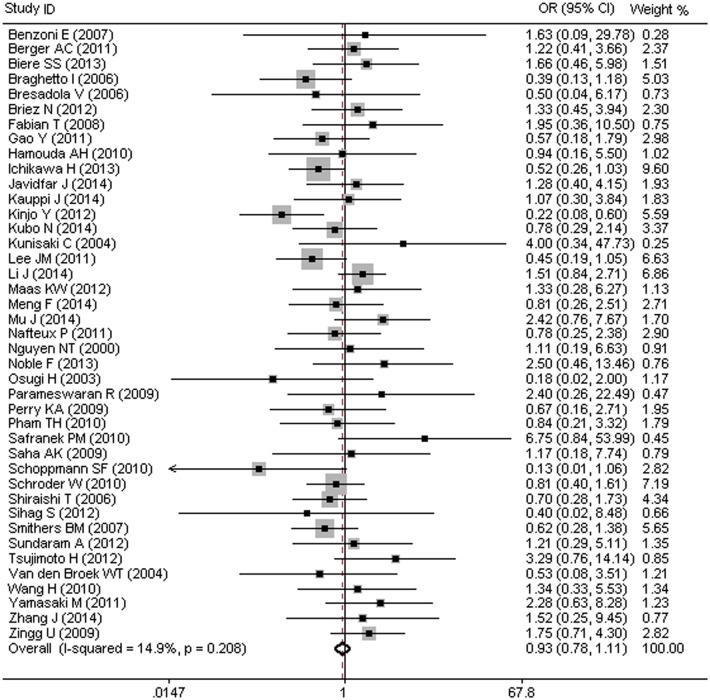
MIO and Risk of Anastomotic Leak (AL).

Forty-one studies carried out on 6,188 patients assessed the effect of MIO on anastomotic leak (AL). Of these patients, 3,152 patients underwent MIO and 3,036 patients underwent OE, with an overall AL morbidity of 9.1% (566/6,188). Fig 6 showed that there was no difference in the occurrence of AL between the MIO and OE groups (OR = 0.93, 95%CI = 0.78–1.11). No heterogeneity was detected among the different studies (I^2^ = 14.9%, P = 0.208).

### MIO and Risk of Gastric Tip Necrosis (GTN)

**Fig 7:**
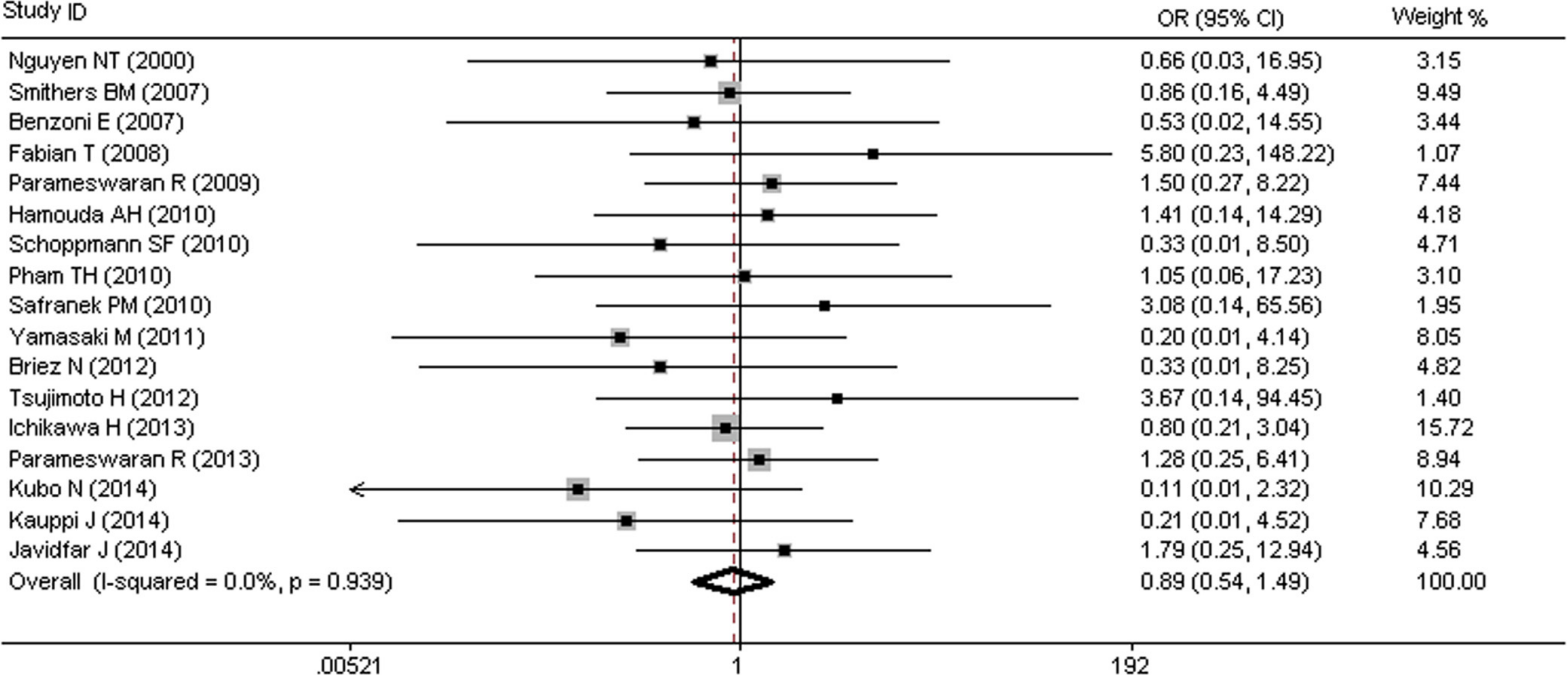
MIO and Risk of Gastric Tip Necrosis (GTN).

Seventeen studies, including a total of 2,570 participants, investigated gastric tip necrosis (GTN) as an outcome measure. Of the included patients, 2.3% (33/1,423) of the patients in MIO group and 2.0% (23/1,147) in OE group developed GTN, with an overall morbidity of 2.2% (56/2,570). The pooled OR 0.89 (95%CI = 0.54–1.49) in Fig 7 showed that no significant difference was found between the two groups, with no heterogeneity (I^2^ = 0.0%, p = 0.939).

### Publication Bias Analysis

Egger's test and Begg’s funnel plots (S1 Fig) were used to assess the publication bias among the included studies. An asymmetric funnel plots figure was shown in S1 Fig, with significantly statistical differences (P<0.05) through Egger's test (S1 Table). This raises the possibility of publication bias. Because of this, we undertook a sensitivity analysis using the trim and fill method, with the aim to impute hypothetically negative unpublished studies to mirror the positive studies that cause funnel plot asymmetry [42]. The pooled analyses after incorporating the hypothetical studies showed consistent results which revealed a statistically significant association between MIO and the risk of IHM.

### Sensitivity Analysis

In the analysis of MIO and risk PCs, sensitivity analyses using the “metaninf” Stata command (S2 Fig) indicated that two independent studies [67, 72], were the main origin of heterogeneity. The heterogeneity was vanished after deletion of the two studies above-mentioned, while the association still kept significant (Fig 3). In addition, no other single study influenced the pooled ORs or RRs qualitatively, as indicated by sensitivity analyses, suggesting that the results of this meta-analysis are stable.

## Discussion

As previously described, minimally invasive oesophagectomy (MIO) has been in existence for almost 20 years and has been used as an option for the curative treatment of esophageal cancer in some centres around the world [28]. Our systematic review and meta-analysis assessed the superiority of MIO in reducing IHM in 14,302 patients from 48 published studies. The major findings of the current meta-analysis provide proof that administration of MIO can significantly decrease the IHM rate in patients with resectable esophageal cancer.

As we have mentioned before, in-hospital mortality (IHM) is an objective, reliable, precise, and bias-free measurement for patients with surgery in hospital databases. The overall IHM rate of 4.0% we found in our meta-analysis was slightly lower than 5% documented in other studies [9, 11]. The underlying reason for the diverse results may be the difference in the surgical procedures for the included patients in the included studies. The pooled OR 0.69 demonstrated that MIO could significantly reduce the risk of IHM, when compared with OE, which was consistent with the results from other studies [4, 68]. The main superiority of MIO over conventional OE was minimal trauma, since in MIO, the operation could be done through small incision, avoiding the trauma of open operation [9]. Moreover, the bias in the selection of patients should be taken into consideration, that is, patients selected for MIO were always in early stages of esophageal cancer, with smaller tumors and lower risk in the occurrence of postoperative complications than patients of late stages.

Pulmonary complications (PCs) are the most frequent morbidity event after oesophagectomy. At least half the patients are at risk for developing PCs after open oesophagectomy performed through a right thoracotomy and laparotomy [9]. In addition, researchers even have reported that the occurrence of PCs are correlated with in-hospital mortality and prolonged hospital stay [6,103]. Therefore, theoretically, we hypothesize that MIO can reduce the rate of PCs and thereby reduce the risk of IHM. The most basic reasons for this hypothesis are the less invasive nature of the procedure and reduced deterioration of the ventilatory mechanism than is observed after the open procedure through avoiding thoracotomy and laparotomy [9,104]. The hypothesis was confirmed in a recently reported multicentre, randomised controlled trial, which found the occurrence of PCs in OE group (29%) was three times more than the MIO group (9%) [3]. In our analysis, when compared to the OE group, the MIO group showed a reduced morbidity of PCs, with the overall PCs morbidity of 19.2% (2,613/13,585). This was consistent with the result of 3.1%-37.0% from other studies [3, 68, 103]. Moreover, the pooled RR of 0.73 indicated that sufficient evidence was available for validation of the superiority of MIO in reducing PCs, with a statistically significant heterogeneity. We also found that two studies reported by Nafteux [67] and Mamidanna [72] due to not taking the TNM stage into account, were the source of heterogeneity. A consistent result from the subgroup analysis after removing the two above- mentioned studies [67, 72] further confirmed the superiority of MIO in reducing the risk of PCs.

We know that cancer patients have a higher risk of deep venous thrombosis (DVT) and even pulmonary embolism (PE) when compared to the general population [105]. And PE has been considered to be associated with arrhythmia, especially atrial fibrillation (AF). Moreover, PE and arrhythmia are recognized as common problems that cause significant morbidity and mortality in modern societies [106]. Hence prevention of postoperative PE and arrhythmia is crucial in reducing the risk of IHM in patients with esophagus carcinoma. Interestingly, it was found in this study that MIO was associated with decreased incidences of PE and arrhythmia. The most fundamental reason for this association is also the less invasive nature of MIO, after which patients easily comply with doctor's advice and start to ambulate as soon as possible. And early ambulation contributes to prevention of thrombosis and thereby prevents the occurrence of PE [107]. In addition, the perforation of minimally invasive surgery per se could decrease the risk factors leading to postoperative cardiac arrhythmia [108]. Therefore, it is reasonable to assume that minimally invasive surgery can mitigate the risk of hospital mortality by reducing the chances of PE and arrhythmia.

Anastomotic leakages (ALs) and gastric tip necrosis (GTN) are fatal complications after oesophagectomyandcan be viewed as catastrophic events [108]. Therefore, the prevention of ALs and GTN appear quite important. In our analysis, no significant difference was found in the occurrence of ALs or GTN between the MIO and OE groups. Such findings indicated that there is still insufficient evidence at present to support the hypothesis that MIO can reduce the occurrence of ALs or GTN for patients with resectable esophageal cancer.

Our meta-analysis has some limitations that might affect the interpretation of the results. First, of the included studies, only one was randomized controlled trial (RCT). The remaining 47 studies used a case-control or cross-sectional design, which is susceptible to recall and selection biases. Therefore, to a certain extent, the included studies cannot provide good evidence for potential treatment effects/ harms, compared to RCTs. Second, the included studies were clinically heterogeneous in some aspects, although the statistical heterogeneity was low. For example, there exists difference in study design, demographics of participants, surgical intervention, operative details, histopathological type, even the outcome reporting after esophageal cancer surgery [103]. Thirdly, we have to emphasize the selection bias in TNM stage of esophageal cancer, that is, patients in MIO group had a higher proportion of early stages when compared to that OE group, although only 31 studies totaling in 4440 cases involved TNM stages. Such bias could result in a lower risk in the occurrence of postoperative complications. However, we were unable to account for these differences, despite the use of appropriate meta-analytic techniques. These limitations may result in an overestimation or underestimation of the effect of MIO. In addition, unmeasured or residual confounding is likely to be present, for instance, the intraoperative collateral tissue damage, bleeding, or worsening organ failure due to surgical trauma.

In conclusion, our research has demonstrated that MIO has superiority in decreasing the incidence of in-hospital mortality, which reinforces the idea that this strategy should be considered as a first-line surgical procedure in esophageal surgery. The decrease in in-hospital mortality by MIO was attributed to the reduction in occurrence of PCs, PE and arrhythmia for patients with resectable esophageal cancer. In addition, more proof is needed for the hypothesis that AL or GTN are two significant contributors to reducing the occurrence of in-hospital mortality.

## Supporting Information

S1 Table Egger's test of interest in Included Studies. (DOCX)

S1 Fig Begg’s funnel plot of In-Hospital Mortality (IHM). (TIF)

S2 Fig Sensitivity Analysis of MIO and risk of PCs. (TIF)

## References

[1] Orringer MB (1984).Transhiatal esophagectomy without thoracotomy for carcinoma of the thoracic esophagus. Ann Surg 200:282–288. 6465981 10.1097/00000658-198409000-00005 PMC1250471

[2] Saito T, Kuwahara A, Hirao E, Shigemitsu Y, Kinoshita T, Kaketani K, et al (1990). Two stages three fields lymph node dissection in esophageal cancer. Nihon Kyobu Geka Gakkai Zasshi 38: 1402–1409. 2246522

[3] Smithers BM, Gotley DC, Martin I, Thomas JM (2007). Comparison of the outcomes between open and minimally invasive esophagectomy. Ann Surg 245:232–240. 17245176 10.1097/01.sla.0000225093.58071.c6 PMC1876975

[4] Biere SS, van Berge Henegouwen MI, Maas KW, Bonavina L, Rosman C, Garcia JR, et al (2012). Minimally invasive versus open oesophagectomy for patients with oesophageal cancer: a multicentre, open-label, randomised controlled trial. Lancet 379:1887–1892. 10.1016/S0140-6736(12)60516-9 22552194

[5] Chen B, Zhang B, Zhu C, Ye Z, Wang C, Ma D, et al (2013). Modified McKeown minimally invasive esophagectomy for esophageal cancer: a 5-year retrospective study of 142 patients in a single institution. PLoS One 8:e82428 10.1371/journal.pone.0082428 24376537 PMC3869695

[6] Kubo N, Ohira M, Yamashita Y, Sakurai K, Toyokawa T, Tanaka H, et al (2014). The Impact of Combined Thoracoscopic and Laparoscopic Surgery on Pulmonary Complications After Radical Esophagectomy in Patients With Resectable Esophageal Cancer. Anticancer Res 34: 2399–2404. 24778050

[7] Morita M, Nakanoko T, Fujinaka Y, Kubo N, Yamashita N, Yoshinaga K, et al (2011). In-hospital mortality after a surgical resection for esophageal cancer: analyses of the associated factors and historical changes. Ann Surg Oncol 18:1757–1765. 10.1245/s10434-010-1502-5 21207167

[8] Hulscher JB, van Sandick JW, de Boer AG, Wijnhoven BP, Tijssen JG, Fockens P, et al (2002). Extended transthoracic resection compared with limited transhiatal resection for adenocarcinoma of the esophagus. N Engl J Med 347:1662–1669. 12444180 10.1056/NEJMoa022343

[9] Luketich JD, Alvelo-Rivera M, Buenaventura PO, Christie NA, McCaughan JS, Litle VR, et al (2003). Minimally invasive esophagectomy: outcomes in 222 patients. Ann Surg 238:486–495. 14530720 10.1097/01.sla.0000089858.40725.68 PMC1360107

[10] Jamieson GG, Mathew G, Ludemann R, Wayman J, Myers JC, Devitt PG (2004). Postoperative mortality following oesophagectomy and problems in reporting its rate. Br J Surg 91:943–947. 15286953 10.1002/bjs.4596

[11] Dimick JB, Staiger DO, Birkmeyer JD (2006). Are mortality rates for different operations related?: implications for measuring the quality of noncardiac surgery. Med Care 44:774–778. 16862040 10.1097/01.mlr.0000215898.33228.c7 PMC2121187

[12] Earlam R, Cunha-Melo JR (1980). Oesophageal squamous cell carcinoma: I. A critical review of surgery. Br J Surg 67:381–390. 6155968 10.1002/bjs.1800670602

[13] Baré M, Cabrol J, Real J, Navarro G, Campo R, Pericay C, et al(2009). In-hospital mortality after stomach cancer surgery in Spain and relationship with hospital volume of interventions. BMC Public Health 9:312 10.1186/1471-2458-9-312 19709446 PMC2749825

[14] Cuschieri A, Shimi S, Banting S(1992). Endoscopic oesophagectomy through a right thoracoscopic approach.J R Coll Surg Edinb 37:7–11. 1573620

[15] Depaula AL, Hashiba K, Ferreira EA, de Paula RA, Grecco E (1995). Laparoscopic transhiatal esophagectomy with esophagogastroplasty. Surg Laparosc Endosc Percut Tech 5:1–5. 7735533

[16] Willer BL, Worrell SG, Fitzgibbons RJ Jr, Mittal SK (2012). Incidence of diaphragmatic hernias following minimally invasive versus open transthoracic Ivor Lewis McKeown esophagectomy. Hernia 16:185–190. 10.1007/s10029-011-0884-z 21983843

[17] Kipfmüller K, Naruhn M, Melzer A, Kessler S, Buess (1989).G.Endoscopic microsurgical dissection of the esophagus.Results in an animal model. Surg Endosc 3:63–69. 2772804 10.1007/BF00590902

[18] Law S (2006).Minimally invasive techniques for oesophageal cancer surgery. Best Pract Res Clin Gastroenterol 20: 925–940. 16997170 10.1016/j.bpg.2006.03.011

[19] Lee L, Sudarshan M, Li C, Latimer E, Fried GM, Mulder DS, et al(2013). Cost-effectiveness of minimally invasive versus open esophagectomy for esophageal cancer. Ann Surg Oncol 20:3732–3739. 10.1245/s10434-013-3103-6 23838923

[20] Yamamoto M, Weber JM, Karl RC, Meredith KL (2013). Minimally invasive surgery for esophageal cancer: review of the literature and institutional experience.Cancer Control 20:130–137. 23571703 10.1177/107327481302000206

[21] Pham TH, Perry KA, Dolan JP, Schipper P, Sukumar M, Sheppard BC, et al (2010). Comparison of perioperative outcomes after combined thoracoscopic-laparoscopic esophagectomy and open Ivor-Lewis esophagectomy. Am J Surg 199:594–598. 10.1016/j.amjsurg.2010.01.005 20466101

[22] Markar SR, Arya S, Karthikesalingam A, anna GB (2013). Technical factors that affect anastomotic integrity following esophagectomy: systematic review and meta-analysis. Ann Surg Oncol 20:4274–4281. 10.1245/s10434-013-3189-x 23943033

[23] Gemmill EH, McCulloch P (2007). Systematic review of minimally invasive resection for gastro-oesophageal cancer. Br J Surg 94:1461–1467. 17973268 10.1002/bjs.6015

[24] Biere SS, Cuesta MA, van der Peet DL (2009).Minimally invasive versus open esophagectomy for cancer: a systematic review and meta-analysis.Minerva Chir 64:121–133. 19365313

[25] Nagpal K, Ahmed K, Vats A, Yakoub D, James D, Ashrafian H, et al (2010). Is minimally invasive surgery beneficial in the management of esophageal cancer? A meta-analysis. Surg Endosc 24:1621–1629. 10.1007/s00464-009-0822-7 20108155

[26] Sgourakis G, Gockel I, Radtke A, Musholt TJ, Timm S, Rink A, et al (2010). Minimally invasive versus open esophagectomy: meta-analysis of outcomes. Dig Dis Sci 55:3031–3040. 10.1007/s10620-010-1153-1 20186484

[27] Butler N, Collins S, Memon B, Memon MA (2011). Minimally invasive oesophagectomy: current status and future direction. Surg Endosc 25:2071–2083 10.1007/s00464-010-1511-2 21298548

[28] Dantoc MM, Cox MR, Eslick GD (2012). Does minimally invasive esophagectomy (MIE) provide for comparable oncologic outcomes to open techniques? A systematic review. J Gastrointest Surg 16:486–494. 10.1007/s11605-011-1792-3 22183862

[29] Dantoc M, Cox MR, Eslick GD (2012). Evidence to support the use of minimally invasive esophagectomy for esophageal cancer: a meta-analysis. Arch Surg 147:768–776. 10.1001/archsurg.2012.1326 22911078

[30] Schumer E, Perry K, Melvin WS (2012). Minimally invasive esophagectomy for esophageal cancer: evolution and review. Surg Laparosc Endosc Percutan Tech 22:383–386. 23047377 10.1097/SLE.0b013e31826295a4

[31] Watanabe M, Baba Y, Nagai Y, Baba H (2013). Minimally invasive esophagectomy for esophageal cancer: an updated review. Surg Today 43:237–244. 10.1007/s00595-012-0300-z 22926551

[32] Uttley L, Campbell F, Rhodes M, Cantrell A, Stegenga H, Lloyd-Jones M (2013). Minimally invasive oesophagectomy versus open surgery: is there an advantage? Surg Endosc 27:724–731. 10.1007/s00464-012-2546-3 23052523

[33] Jacobs M, Macefield RC, Elbers RG, Sitnikova K, Korfage IJ, Smets EM, et al (2014). Meta-analysis shows clinically relevant and long-lasting deterioration in health-related quality of life after esophageal cancer surgery. Qual Life Res 23:1155–1176. 10.1007/s11136-013-0576-5 24293086

[34] Booth A, Clarke M, Dooley G, Ghersi D, Moher D, Petticrew M, et al (2012). The nuts and bolts of PROSPERO: an international prospective register of systematic reviews. Systematic Reviews 1:1–8. 22587842 10.1186/2046-4053-1-2 PMC3348673

[35] Moher D, Liberati A, Tetzlaff J, Altman DG; PRISMA Group (2009). Preferred reporting items for systematic reviews and meta-analyses: the PRISMA statement. Ann Intern Med 151: 264–269. 19622511 10.7326/0003-4819-151-4-200908180-00135

[36] Liberati A, Altman DG, Tetzlaff J, Mulrow C, Gøtzsche PC, Ioannidis JP, et al (2009).The PRISMA statement for reporting systematic reviews and meta-analyses of studies that evaluate health care interventions:explanation and elaboration. PLoS Med 6:e1000100 10.1371/journal.pmed.1000100 19621070 PMC2707010

[37] Slim K, Nini E, Forestier D, Kwiatkowski F, Panis Y, Chipponi J (2003).Methodological index for non-randomized studies (MINORS): development and validation of a new instrument. ANZ J Surg 73:712–716. 12956787 10.1046/j.1445-2197.2003.02748.x

[38] DerSimonian R, Laird N (1986).Meta-analysis in clinical trials. Control Clin Trials 7:177–188. 3802833 10.1016/0197-2456(86)90046-2

[39] Higgins JP, Thompson SG, Deeks JJ, Altman DG (2003). Measuring inconsistency in meta-analyses. BMJ 327:557–560. 12958120 10.1136/bmj.327.7414.557 PMC192859

[40] Higgins JP, Thompson SG (2002). Quantifying heterogeneity in a meta-analysis. Stat Med 21: 1539–1558. 12111919 10.1002/sim.1186

[41] Egger M, Davey Smith G, Minder C (1997). Bias in meta-analysis detected by a simple, graphical test. BMJ 315: 629–634; 9310563 10.1136/bmj.315.7109.629 PMC2127453

[42] Willi C, Bodenmann P, Ghali WA, Faris PD, Cornuz J (2007). Active smoking and the risk of type 2 diabetes: a systematic review and meta-analysis. JAMA 298:2654–2664. 18073361 10.1001/jama.298.22.2654

[43] Law S, Fok M, Chu KM, Wong J (1997). Thoracoscopic esophagectomy for esophageal cancer. Surgery 122:8–14. 9225908 10.1016/s0039-6060(97)90257-9

[44] Nguyen NT, Follette DM, Wolfe BM, Schneider PD, Roberts P, Goodnight JE Jr (2000). Comparison of minimally invasive esophagectomy with transthoracic and transhiatal esophagectomy. Arch Surg 135:920–925. 10922253 10.1001/archsurg.135.8.920

[45] Osugi H, Takemura M, Higashino M, Takada N, Lee S, Kinoshita H (2003). A comparison of video-assisted thoracoscopic oesophagectomy and radical lymph node dissection for squamous cell cancer of the oesophagus with open operation. Br J Surg 90:108–113. 12520585 10.1002/bjs.4022

[46] Kunisaki C, Hatori S, Imada T, Akiyama H, Ono H, Otsuka Y, et al (2009) Video-assisted Thoracoscopic Esophagectomy With a Voice-controlled Robot: The AESOP System. Langenbecks Arch Surg 394:617–621. 15599295 10.1097/01.sle.0000148468.74546.9a

[47] Van den Broek WT, Makay O, Berends FJ, Yuan JZ, Houdijk AP, Meijer S, et al (2004). Laparoscopically assisted transhiatal resection for malignancies of the distal esophagus. Surg Endosc 18: 812–817. 15216864 10.1007/s00464-003-9173-y

[48] Braghetto I, Csendes A, Cardemil G, Burdiles P, Korn O, Valladares H (2006).Open transthoracic or transhiatal esophagectomy versus minimally invasive esophagectomy in terms of morbidity, mortality and survival.Surg Endosc 20: 1681–1686. 16960662 10.1007/s00464-006-0009-4

[49] Bresadola V, Terrosu G, Cojutti A, Benzoni E, Baracchini E, Bresadola F (2006).Surg Laparosc Endosc Percutan Tech. Laparoscopic Versus Open Gastroplasty in Esophagectomy for Esophageal Cancer: A Comparative Study. Surg Laparosc Endosc Percutan Tech 16: 63–67. 16773002 10.1097/00129689-200604000-00001

[50] Shiraishi T, Kawahara K, Shirakusa T, Yamamoto S, Maekawa T (2006). Risk analysis in resection of thoracic esophageal cancer in the era of endoscopic surgery. Ann Thorac Surg 81: 1083–1089. 16488728 10.1016/j.athoracsur.2005.08.057

[51] Benzoni E, Terrosu G, Bresadola V, Uzzau A, Intini S, Noce L (2007).A comparative study of the transhiatal laparoscopic approach versus laparoscopic gastric mobilisation and right open transthoracic esophagectomy for esophageal cancer management.J Gastrointestin Liver Dis 16:395–401. 18193121

[52] Fabian T, Martin JT, McKelvey AA, Federico JA (2008).Minimally invasive esophagectomy: a teaching hospital's first year experience.Dis Esophagus 21:220–225. 10.1111/j.1442-2050.2007.00783.x 18430102

[53] Kitagawa H, Akimori T, Okabayashi T, Namikawa T, Sugimoto T, Kobayashi M, et al (2009). Total laparoscopic gastric mobilization for esophagectomy. Langenbecks Arch Surg 394:617–621. 10.1007/s00423-008-0354-y 18542990

[54] Parameswaran R, Veeramootoo D, Krishnadas R, Cooper M, Berrisford R, Wajed S (2009). Comparative Experience of Open and Minimally Invasive Esophagogastric Resection. World J Surg 33:1868–1875. 10.1007/s00268-009-0116-1 19609827

[55] Perry KA, Enestvedt CK, Pham T, Welker M, Jobe BA, Hunter JG, et al (2009).Comparison of Laparoscopic Inversion Esophagectomy and Open Transhiatal Esophagectomy for High-Grade Dysplasia and Stage I Esophageal Adenocarcinoma. Arch Surg 144:679–684. 10.1001/archsurg.2009.113 19620549

[56] Saha AK, Sutton CD, Sue-Ling H, Dexter SP, arela AI (2009). Comparison of oncological outcomes after laparoscopic transhiatal and open esophagectomy for T1 esophageal adenocarcinoma. Surg Endosc 23:119–124. 10.1007/s00464-008-0065-z 18626700

[57] Zingg U, McQuinn A, DiValentino D, Esterman AJ, Bessell JR, Thompson SK, et al (2009). Minimally invasive versus open esophagectomy for patients with esophageal cancer. Ann Thorac Surg 87: 911–919. 10.1016/j.athoracsur.2008.11.060 19231418

[58] Hamouda AH, Forshaw MJ, Tsigritis K, Jones GE, Noorani AS, Rohatgi A (2010). Perioperative outcomes after transition from conventional to minimally invasive Ivor-Lewis esophagectomy in a specialized center.Surg Endosc 24:865–869. 10.1007/s00464-009-0679-9 19730947

[59] Safranek PM, Cubitt J, Booth MI, Dehn TC (2010). Review of open and minimal access approaches to oesophagectomy for cancer. Br J Surg 97:1845–1853. 10.1002/bjs.7231 20922782

[60] Schoppmann SF, Prager G, Langer FB, Riegler FM, Kabon B, Fleischmann E, et al (2010).Open versus minimally invasive esophagectomy: a single-center case controlled study. Surg Endosc 24: 3044–3053. 10.1007/s00464-010-1083-1 20464423

[61] Schröder W, Hölscher AH, Bludau M, Vallböhmer D, Bollschweiler E, Gutschow C (2010). Ivor-Lewis esophagectomy with and without laparoscopic conditioning of the gastric conduit. World J Surg 34:738–743. 10.1007/s00268-010-0403-x 20098986

[62] Tsujimoto H, Ono S, Sugasawa H, Ichikura T, Yamamoto J, Hase K (2010).Gastric tube reconstruction by laparoscopy-assisted surgery attenuates postoperative systemic inflammatory response after esophagectomy for esophageal cancer. World J Surg 34:2830–2836. 10.1007/s00268-010-0757-0 20703457

[63] Wang H, Feng M, Tan L, Wang Q (2010). Comparison of the short-term quality of life in patients with esophageal cancer after subtotal esophagectomy via video-assisted thoracoscopic or open surgery. Dis Esophagus 23:408–414. 10.1111/j.1442-2050.2009.01025.x 19930404

[64] Berger AC, Bloomenthal A, Weksler B, Evans N, Chojnacki KA, Yeo CJ, et al (2011). Oncologic efficacy is not compromised, and may be improved with minimally invasive esophagectomy. J Am Coll Surg 212:560–566. 10.1016/j.jamcollsurg.2010.12.042 21463789

[65] Gao Y, Wang Y, Chen L, Zhao Y (2011). Comparison of open three-field and minimally-invasive esophagectomy for esophageal cancer.Interact Cardiovasc Thorac Surg 12:366–369. 10.1510/icvts.2010.258632 21186282

[66] Lee JM, Cheng JW, Lin MT, Huang PM, Chen JS, Lee YC(2011).Is there any benefit to incorporating a laparoscopic procedure into minimally invasive esophagectomy? The impact on perioperative results in patients with esophageal cancer. World J Surg 35:790–797. 10.1007/s00268-011-0955-4 21327605

[67] Nafteux P, Moons J, Coosemans W, Decaluwé H, Decker G, De Leyn P, et al (2011). Minimally invasive oesophagectomy: a valuable alternative to open oesophagectomy for the treatment of early oesophageal and gastro-oesophageal junction carcinoma. Eur J Cardiothorac Surg 40:1455–1463. discussion 1463–1464. 10.1016/j.ejcts.2011.01.086 21514837

[68] Yamasaki M, Miyata H, Fujiwara Y, Takiguchi S, Nakajima K, Kurokawa Y, et al (2011). Minimally invasive esophagectomy for esophageal cancer: comparative analysis of open and hand-assisted laparoscopic abdominal lymphadenectomy with gastric conduit reconstruction.J Surg Oncol. 104: 623–628; 10.1002/jso.21991 21695699

[69] Briez N, Piessen G, Torres F, Lebuffe G, Triboulet JP, Mariette C(2012).Effects of hybrid minimally invasive oesophagectomy on major postoperative pulmonary complications. Br J Surg 99: 1547–1553. 10.1002/bjs.8931 23027071

[70] Kinjo Y, Kurita N, Nakamura F, Okabe H, Tanaka E, Kataoka Y, et al (2012). Effectiveness of combined thoracoscopic-laparoscopic esophagectomy: comparison of postoperative complications and midterm oncological outcomes in patients with esophageal cancer. Surg Endosc 26:381–389. 10.1007/s00464-011-1883-y 21898014

[71] Maas KW, Biere SS, Scheepers JJ, Gisbertz SS, van-der-Peet DL, Cuesta MA (2012). Laparoscopic versus open transhiatal esophagectomy for distal and junction cancer. Rev Esp Enferm Dig 104:197–202. 22537368 10.4321/s1130-01082012000400005

[72] Mamidanna R, Bottle A, Aylin P, Faiz O, Hanna GB (2012). Short-term outcomes following open versus minimally invasive esophagectomy for cancer in England: a population-based national study. Ann Surg 255:197–203; 22173202 10.1097/SLA.0b013e31823e39fa

[73] Sihag S, Wright CD, Wain JC, Gaissert HA, Lanuti M, Allan JS, et al (2012).Comparison of perioperative outcomes following open versus minimally invasive Ivor Lewis oesophagectomy at a single,high-volume centre. Eur J Cardiothorac Surg 42:430–437. 10.1093/ejcts/ezs031 22345284

[74] Sundaram A, Geronimo JC, Willer BL, Hoshino M, Torgersen Z, Juhasz A, et al (2012).Survival and quality of life after minimally invasive esophagectomy: a single-surgeon experience. Surg Endosc 26:168–176. 10.1007/s00464-011-1850-7 21853394

[75] Tsujimoto H, Takahata R, Nomura S, Yaguchi Y, Kumano I, Matsumoto Y, et al(2012). Video-assisted thoracoscopic surgery for esophageal cancer attenuates postoperative systemic responses and pulmonary complications. Surgery 151:667–673. 10.1016/j.surg.2011.12.006 22244180

[76] Bailey L, Khan O, Willows E, Somers S, Mercer S, Toh S(2013). Open and laparoscopically assisted oesophagectomy: a prospective comparative study. Eur J Cardiothorac Surg 43:268–273. 10.1093/ejcts/ezs314 22753051

[77] Ichikawa H, Miyata G, Miyazaki S, Onodera K, Kamei T, Hoshida T, et al (2013). Esophagectomy using a thoracoscopic approach with an open laparotomic or hand-assisted laparoscopic abdominal stage for esophageal cancer: analysis of survival and prognostic factors in 315 patients.Ann Surg 257:873–885. 23001081 10.1097/SLA.0b013e31826c87cd

[78] Kitagawa H, Namikawa T, Iwabu J, Akimori T, Okabayashi T, Sugimoto T, et al (2013). Efficacy of laparoscopic gastric mobilization for esophagectomy: comparison with open thoraco-abdominal approach. J Laparoendosc Adv Surg Tech A 23:452–455. 10.1089/lap.2012.0377 23560659

[79] Noble F, Kelly JJ, Bailey IS, Byrne JP, Underwood TJ; South Coast Cancer Collaboration—Oesophago-Gastric (SC3-OG) (2013).A prospective comparison of totally minimally invasive versus open Ivor Lewis esophagectomy. Dis Esophagus 26:263–271. 10.1111/j.1442-2050.2012.01356.x 23551569

[80] Parameswaran R, Titcomb DR, Blencowe NS, Berrisford RG, Wajed SA, Streets CG, et al(2013). Assessment and comparison of recovery after open and minimally invasive esophagectomy for cancer: an exploratory study in two centers. Ann Surg Oncol 20:1970–1977. 10.1245/s10434-012-2848-7 23306956

[81] Takeno S, Takahashi Y, Moroga T, Kawahara K, Yamashita Y, Ohtaki M (2013). Retrospective study using the propensity score to clarify the oncologic feasibility of thoracoscopic esophagectomy in patients with esophageal cancer. World J Surg 37:1673–1680. 10.1007/s00268-013-2008-7 23539192

[82] Mu J, Yuan Z, Zhang B, Li N, Lyu F, Mao Y, et al(2014). Comparative study of minimally invasive versus open esophagectomy for esophageal cancer in a single cancer center. Chin Med J (Engl) 127: 747–752. 24534234

[83] Kauppi J, Räsänen J, Sihvo E, Huuhtanen R, Nelskylä K, Salo J (2014). Open versus minimally invasive esophagectomy: clinical outcomes for locally advanced esophageal adenocarcinoma. Surg Endosc Dec 6. [Epub ahead of print] 10.1007/s00464-014-3978-8 25480610

[84] Meng F, Li Y, Ma H, Yan M, Zhang R (2014). Comparison of outcomes of open and minimally invasive esophagectomy in 183 patients with cancer. J Thorac Dis 6:1218–1224. 10.3978/j.issn.2072-1439.2014.07.20 25276363 PMC4178093

[85] Schneider C, Boddy AP, Fukuta J, Groom WD, Streets CG (2014). Predicting blood transfusion in patients undergoing minimally invasive oesophagectomy. Int J Surg 12:1342–1347. 10.1016/j.ijsu.2014.10.016 25448656

[86] Zhang J, Xu M, Guo M, Mei X, Liu C (2014). Analysis of postoperative quality of life in patients with middle thoracic esophageal carcinoma undergoing minimally invasive Ivor-Lewis esophagectomy. Zhonghua Wei Chang Wai Ke Za Zhi 17:915–919. 25273663

[87] Li J, Shen Y, Tan L, Feng M, Wang H, Xi Y, et al (2015).Is minimally invasive esophagectomy beneficial to elderly patients with esophageal cancer. Surg Endosc 29:925–930. 10.1007/s00464-014-3753-x 25249141

[88] Javidfar J, Bacchetta M, Yang JA, Miller J, D'Ovidio F, Ginsburg ME, et al (2012). The use of a tailored surgical technique for minimally invasive esophagectomy. J Thorac Cardiovasc Surg 143: 1125–1129. 10.1016/j.jtcvs.2012.01.071 22500593

[89] Briez N, Piessen G, Bonnetain F, Brigand C, Carrere N, Collet D, et al (2011). Open versus laparoscopically-assisted oesophagectomy for cancer: a multicentre randomised controlled phase III trial—the MIRO trial. BMC Cancer 11:310 10.1186/1471-2407-11-310 21781337 PMC3156811

[90] Willer BL, Worrell SG, Fitzgibbons RJ Jr, Mittal SK (2012). Incidence of diaphragmatic hernias following minimally invasive versus open transthoracic Ivor Lewis McKeown esophagectomy. Hernia 16:185–190. 10.1007/s10029-011-0884-z 21983843

[91] Khan O, Nizar S, Vasilikostas G, Wan A (2012). Minimally invasive versus open oesophagectomy for patients with oesophageal cancer: a multicentre, open-label, randomised controlled trial. J Thorac Dis 4:465–466. 10.3978/j.issn.2072-1439.2012.08.16 23050109 PMC3461074

[92] Cuesta MA, Biere SS, Henegouwen MI, van der Peet DL (2012). Randomised trial, Minimally Invasive Oesophagectomy versus open oesophagectomy for patients with resectable oesophageal cancer. J Thorac Dis 4:462–464. 10.3978/j.issn.2072-1439.2012.08.12 23050108 PMC3461077

[93] Veeramootoo D, Shore AC, Wajed SA (2012). Randomized controlled trial of laparoscopic gastric ischemic conditioning prior to minimally invasive esophagectomy, the LOGIC trial. Surg Endosc 26:1822–1829. 10.1007/s00464-011-2123-1 22302533

[94] Lee L, Sudarshan M, Li C, Latimer E, Fried GM, Mulder DS, et al (2013). Cost-effectiveness of minimally invasive versus open esophagectomy for esophageal cancer. Ann Surg Oncol 20:3732–3739. 10.1245/s10434-013-3103-6 23838923

[95] Avery KN, Metcalfe C, Berrisford R, Barham CP, Donovan JL, Elliott J, et al (2014). The feasibility of a randomized controlled trial of esophagectomy for esophageal cancer—the ROMIO (Randomized Oesophagectomy: Minimally Invasive or Open) study: protocol for a randomized controlled trial. Trials 15:200 10.1186/1745-6215-15-200 24888266 PMC4084574

[96] Anderegg MC, Gisbertz SS, van Berge Henegouwen MI (2014). Minimally invasive surgery for oesophageal cancer. Best Pract Res Clin Gastroenterol 28:41–52. 10.1016/j.bpg.2013.11.002 24485254

[97] Bernabe KQ, Bolton JS, Richardson WS (2005). Laparoscopic hand-assisted versus open transhiatalesophagectomy: a case-control study.SurgEndosc 19: 334–337. 10.1007/s00464-004-8807-z 15959707

[98] Thomson IG, Smithers BM, Gotley DC, Martin I, Thomas JM, O'Rourke P, et al (2010).Thoracoscopic-assisted esophagectomy for esophageal cancer: analysis of patterns and prognostic factors for recurrence. Ann Surg 252:281–291. 20647926 10.1097/SLA.0b013e3181e909a2

[99] Seng C, Siddiqui MA, Wong KP, Zhang K, Yeo W, Tan SB, et al(2013). Five-year outcomes of minimally invasive versus open transforaminal lumbar interbody fusion: a matched-pair comparison study. Spine (Phila Pa 1976) 38:2049–2055. 23963015 10.1097/BRS.0b013e3182a8212d

[100] Taguchi S, Osugi H, Higashino M, Tokuhara T, Takada N, Takemura M, et al (2003). Comparison of three-field esophagectomy for esophageal cancer incorporating open or thoracoscopic thoracotomy. Surg Endosc 17:1445–1450. 12811660 10.1007/s00464-002-9232-9

[101] Scheepers JJ, van der Peet DL, Veenhof AA, Cuesta MA (2009). Influence of circumferential resection margin on prognosis in distal esophageal and gastroesophageal cancer approached through the transhiatal route. Dis Esophagus 22:42–48. 10.1111/j.1442-2050.2008.00898.x 19196265

[102] Burdall OC, Boddy AP, Fullick J, Blazeby J, Krysztopik R, Streets C, et al (2015). A comparative study of survival after minimally invasive and open oesophagectomy. Surg Endosc 29:431–437. 10.1007/s00464-014-3694-4 25125095

[103] Ferguson MK, Celauro AD, Prachand V (2011). Prediction of major pulmonary complications after esophagectomy. Ann Thorac Surg 91:1494–1500; discussion 1500–1501. 10.1016/j.athoracsur.2010.12.036 21524462

[104] Korst RJ, Port JL, Lee PC, Altorki NK (2005). Intrathoracic manifestations of cervical anastomotic leaks after transthoracic esophagectomy for carcinoma. Ann Thorac Surg 80:1185–1190. 16181838 10.1016/j.athoracsur.2005.04.020

[105] Gex G, Gerstel E, Righini M, LE Gal G, Aujesky D, Roy PM, et al(2012). Is atrial fibrillation associated with pulmonary embolism? J Thromb Haemost 10:347–351. 10.1111/j.1538-7836.2011.04608.x 22212132

[106] Trujillo-Santos J, Perea-Milla E, Jiménez-Puente A, Sánchez-Cantalejo E, del Toro J, Grau E, et al (2005). Bed rest or ambulation in the initial treatment of patients with acute deep vein thrombosis or pulmonary embolism: findings from the RIETE registry. Chest 127:1631–1636. 15888839 10.1378/chest.127.5.1631

[107] Xue L, Pan T, Xu Z, Zhao X, Zhong L, Wu L, et al (2009).Multi-factor investigation of early postoperative cardiac arrhythmia for elderly patients with esophageal or cardiac carcinoma. World J Surg 33:2615–2619. 10.1007/s00268-009-0222-0 19760310

[108] Weidenhagen R, Hartl WH, Gruetzner KU, Eichhorn ME, Spelsberg F, Jauch KW (2010). Anastomotic leakage after esophageal resection: new treatment options by endoluminal vacuum therapy. Ann Thorac Surg 90:1674–1681. 10.1016/j.athoracsur.2010.07.007 20971288

