# Supplementary material for: Superiority of Minimally Invasive Oesophagectomy in Reducing In-Hospital Mortality of Patients with Resectable Oesophageal Cancer: A Meta-Analysis
Source: PLoS One. 2015 Jul 21;10(7):e0132889. doi: 10.1371/journal.pone.0132889 (PMC4509855; doi:10.1371/journal.pone.0132889)
Supplement: S1 Table — (DOCX) [file pone.0132889.s001.docx]

S1Table Egger's test of interest in Included Studies

| Std_Eff | IHM | |
| --- | --- | --- |
|  | slope | bias |
| Coef. | -0.07041 | -0.50913 |
| Std. Err. | 0.132764 | 0.207476 |
| t | -0.53000 | -2.45000 |
| P>t | 0.60000 | 0.02000 |
| 95% CI. |  |  |
| Lower | -0.34194 | -0.93346 |
| Upper | 0.201125 | -0.08479 |
