# Supplementary figures and images for: Superiority of Minimally Invasive Oesophagectomy in Reducing In-Hospital Mortality of Patients with Resectable Oesophageal Cancer: A Meta-Analysis
Source: PLoS One. 2015 Jul 21;10(7):e0132889. doi: 10.1371/journal.pone.0132889 (PMC4509855; doi:10.1371/journal.pone.0132889)

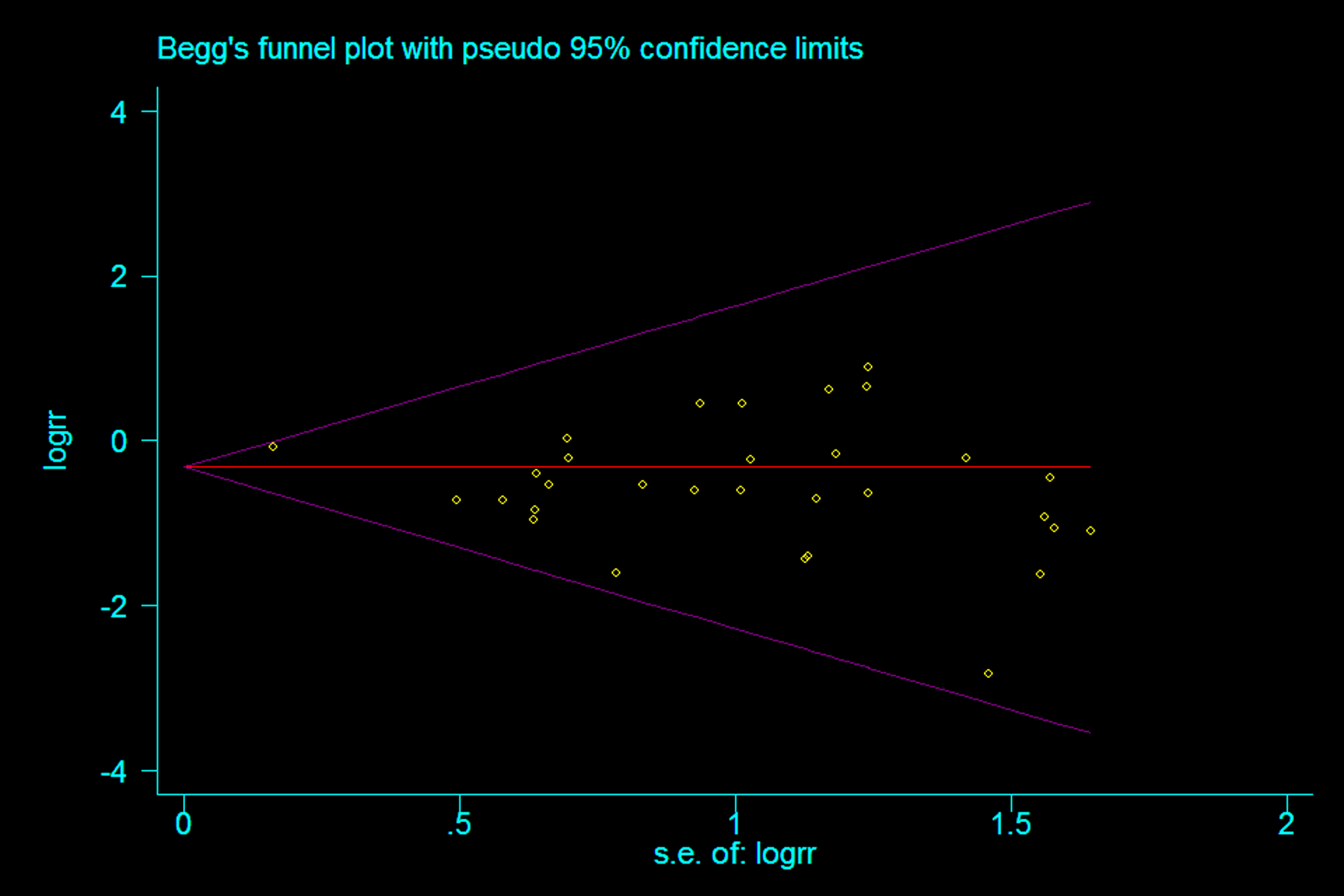

Supplement: S1 Fig — (TIF) [file pone.0132889.s002.tif]

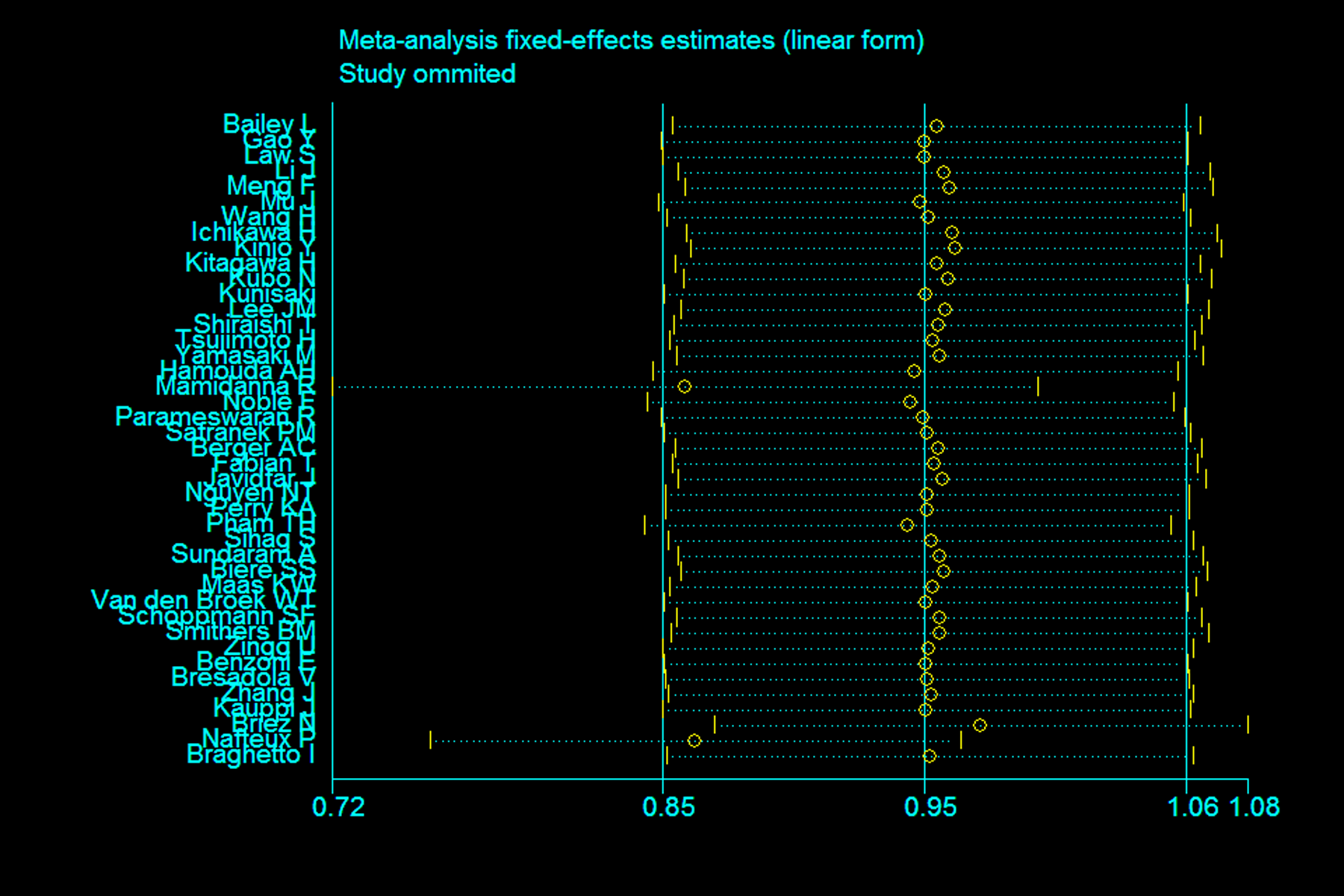

Supplement: S2 Fig — (TIF) [file pone.0132889.s003.tif]
